# Hypoxia-sensitive long noncoding RNA CASC15 promotes lung tumorigenesis by regulating the SOX4/β-catenin axis

**DOI:** 10.1186/s13046-020-01806-5

**Published:** 2021-01-06

**Authors:** Jianyong Sun, Yanlu Xiong, Kuo Jiang, Bo Xin, Tongtong Jiang, Renji Wei, Yuankang Zou, Hong Tan, Tao Jiang, Angang Yang, Lintao Jia, Lei Wang

**Affiliations:** 1 State Key Laboratory of Cancer Biology, Department of Biochemistry and Molecular Biology, Fourth Military Medical University, Xi’an, 710032 Shaanxi China; 2 Department of Thoracic Surgery, Tangdu Hospital, Fourth Military Medical University, Xi’an, 710038 Shaanxi China; 3 Department of Spine Surgery, Honghui Hospital, Xi’an Jiaotong University, Xi’an, 710054 Shaanxi China; 4 Department of Oncology, The 960th Hospital of PLA, Tai’an, 271000 Shandong China; 5 The Ministry of Education Key Lab of Hazard Assessment and Control in Special Operational Environment, Department of Occupational and Environmental Health, Fourth Military Medical University, Xi’an, 710032 Shaanxi China; 6 Department of Immunology, Fourth Military Medical University, Xi’an, 710032 Shaanxi China

**Keywords:** Long noncoding RNA, CASC15, SOX4, Non-small cell lung cancer, Hypoxia signaling

## Abstract

**Background:**

Accumulating evidence has demonstrated that long non-coding RNAs (lncRNAs) are involved in the hypoxia-related cancer process and play pivotal roles in enabling malignant cells to survive under hypoxic stress. However, the molecular crosstalk between lncRNAs and hypoxia signaling cascades in non-small cell lung cancer (NSCLC) remains largely elusive.

**Methods:**

Firstly, we identified differentially expressed lncRNA cancer susceptibility candidate 15 (CASC15) as associated with NSCLC based on bioinformatic data. The clinical significance of CASC15 in lung cancer was investigated by Kaplan-Meier survival analysis. Then, we modulated CASC15 expression in NSCLC cell lines by RNAi. CCK-8 and transwell assays were carried out to examine the effects of CASC15 on proliferation and migration of NSCLC cells. Upstream activator and downstream targets of CASC15 were validated by luciferase reporter assay, qRT-PCR, Western blotting, and chromatin immunoprecipitation (ChIP). Lastly, RNA in situ hybridization (RNA-ISH) and immunohistochemistry (IHC) were performed to confirm the genetic relationships between CASC15 and related genes in clinical samples.

**Results:**

CASC15 was highly expressed in NSCLC tissues and closely associated with poor prognosis. Loss-of-function analysis demonstrated that CASC15 was essential for NSCLC cell migration and growth. Mechanistic study revealed that CASC15 was transcriptionally activated by hypoxia signaling in NSCLC cells. Further analysis showed that hypoxia-induced CASC15 transactivation was mainly dependent on hypoxia-inducible factor 1α (HIF-1α) and hypoxia response elements (HREs) located in *CASC15* promoter. CASC15 promotes the expression of its chromosomally nearby gene, SOX4. Then SOX4 functions to stabilize β-catenin protein, thereby enhancing the proliferation and migration of NSCLC cells. HIF-1α/CASC15/SOX4/β-catenin pathway was activated in a substantial subset of NSCLC patients.

**Conclusions:**

HIF-1α/CASC15/SOX4/β-catenin axis plays an essential role in the development and progression of NSCLC. The present work provides new evidence that lncRNA CASC15 holds great promise to be used as novel biomarkers for NSCLC. Blocking the HIF-1α/CASC15/SOX4/β-catenin axis can serve as a potential therapeutic strategy for treating NSCLC.

## Background

Lung cancer is the leading cause of cancer incidence and mortality around the world, with 2.1 million new lung cancer cases and 1.8 million deaths predicted in 2018, representing close to 1 in 5 (18.4%) cancer deaths [1]. In China, lung cancer incidence in both men and women has increased rapidly in recent years, imposing a great threat to human health [2]. Non-small cell lung cancer (NSCLC) accounts for approximately 85% of all lung cancers, and adenocarcinoma and squamous cell carcinoma are two major histologic subtypes of NSCLC [3]. Despite recent advances in surgery, chemotherapy and radiation therapy, the overall 5-year survival rate for NSCLC patients remains as low as 15.9% [4]. Thus, a better understanding of the underlying mechanisms and molecular pathways in NSCLC development and progression is particularly important for the precise treatment of NSCLC.

SOX4 (SRY-Box Transcription Factor 4) is a member of the group C subfamily of the SOX transcription factors and plays critical roles in numerous aspects of embryogenesis. It is widely expressed in developing embryos, but in adults, SOX4 expression is found only in a limited set of tissues [5]. SOX4 expression is elevated in a large variety of tumor types, including leukemia, colorectal cancer, breast cancer and lung cancer, suggesting a fundamental role in the development of these malignancies [6]. Activation of SOX4 could affect many distinct biological processes, such as inhibition of apoptosis, enhanced cell migration and metastasis, and the induction and maintenance of tumor-initiating cells (TICs) [6]. Several cancer-associated signaling pathways have been implicated in the activation of SOX4, including Wnt, TNF-α, TGF-β, and hypoxia/HIF-1α signaling [7]. In the particular case of lung cancer, SOX4 was found to be overexpressed partially due to genetic amplification of the *SOX4* locus [8], and increased expression of SOX4 was identified as a potential biomarker for tumor malignancy and poor prognosis in patients with NSCLC [9]. Moreover, global expression analysis performed in lung cancer confirmed upregulation of the SOX4 expression signature in a panel of primary lung tumors [10]. However, genomic amplification accounted for the upregulation of SOX4 in only a minority of lung carcinomas (9%, 4 out of 42), [8], and the detailed molecular mechanisms underlying SOX4 overexpression in NSCLC remain largely unknown.

Long noncoding RNAs (lncRNAs) are a large heterogeneous class of transcripts longer than 200 nucleotides with limited protein-coding potential [11]. Recent evidence has demonstrated that lncRNAs are pervasively transcribed throughout eukaryotic genomes, implicating their significant regulatory roles in complex organisms [12, 13]. Although only a small portion of functional lncRNAs have been well characterized to date, they have been shown to control nearly every aspect of gene expression and a diverse set of cellular processes, such as cell proliferation and migration [14, 15]. Recently, several studies reported that lncRNAs represent some of the most differentially expressed transcripts between lung tumor and normal lung tissues [16, 17], highlighting their potential in lung cancer initiation and progression. In this work, we found that one lncRNA, annotated as CASC15 (cancer susceptibility candidate 15) and located in the neighbourhood of the *SOX4* locus, could upregulate SOX4 expression in NSCLC cell lines. The expression of CASC15 is elevated in NSCLC samples compared with normal lung tissues and is positively correlated with that of SOX4. Mechanistically, CASC15 is transcriptionally activated by the hypoxia/HIF-1α signaling and promotes SOX4 expression in a *cis*-acting manner. SOX4 functions to stabilize β-catenin protein, thereby enhancing the proliferation and migration of NSCLC cells. The present study demonstrated for the first time that the HIF-1α/CASC15/SOX4/β-catenin axis plays an essential role in the development and progression of NSCLC, and lncRNA CASC15 holds great promise to be used as novel biomarkers and therapeutic targets for NSCLC.

## Methods

### Cell culture and transfection

Five NSCLC cell lines (A549, H1299, H1650, H1975, H520) and a normal lung bronchus epithelial cell line BEAS-2B were obtained from American Type Culture Collection (ATCC; Rockville, MD, USA). NSCLC cells were cultured in RPMI 1640 (Gibco, Grand Island, NY) medium supplemented with 10% fetal bovine serum, in humidified air at 37 °C with 5% CO_2_. BEAS-2B cells were cultured in serum-free LHC-9 medium (Biofluids, Rockville, MD). Transfection was performed using Lipofectamine 2000 reagent (Invitrogen). si-CASC15, si-SOX4, si-HIF1A were purchased from GenePharma (Shanghai, China). For lentiviral transduction of NSCLC cells, cells were infected with recombinant lentiviruses and were selected with puromycin (1 μg/mL) or blasticidin (10 μg/mL) for 7 days prior to use of homogenous pool of the infected cells for further assays. Lentiviruses carrying CASC15-targeting shRNA or SOX4 cDNA were purchased from GeneChem (Shanghai, China).

### Patients and specimens

Fresh frozen primary NSCLC tissues and adjacent non-tumorous lung tissues were obtained from Chinese patients at Tangdu Hospital (Xi’an, China). Tissue microarray blocks consisted of 35 matched pairs of NSCLC and adjacent normal lung tissues were purchased from Servicebio (Wuhan, China). Clinical characterizations of NSCLC patients are presented in Table S1. The use of clinical specimens and commercially obtained samples in this study was approved by the Tangdu Hospital Ethic Committee in Fourth Military Medical University.

### Mice and tumor models

Six-week-old male nude mice were purchased from the Model Animal Research Center of Nanjing University (Nanjing, China) and maintained under specific pathogen-free conditions. Mice had free access to food and water during the whole experimental period. NSCLC cell lines stably expressing luciferase gene are routinely cultivated in our laboratory, and 5 × 10^6^ cells were injected into nude mice subcutaneously. Tumor growth were assessed weekly by bioluminescent imaging on the Xenogen In Vivo Imaging System (IVIS, Caliper Life Science, Hopkinton, MA). All animal experiments were approved by the Institutional Animal Care and Use Committee of Fourth Military Medical University.

### Bioinformatics analysis

Gene expression profiling and clinical data for NSCLC patients were obtained from the GEO and TCGA databases. The GDS3837 database, which included sixty pairs of human lung carcinomas and adjacent normal lung tissues, was used to investigate CASC15 expression levels. The GSE30219 database, which included 293 lung tumor tissues, was used to investigate the relationship between overall survival and CASC15 expression levels. The TCGA-LUAD cohort, which included 515 lung adenocarcinoma tissues, was used to investigate the correlation between CASC15 expression and HIF1A/SOX4/CTNNB1 expression levels. The significance of pairwise correlations among CASC15, HIF1A, SOX4 and CTNNB1 expression levels in TCGA-LUAD cohort was judged via a test statistic based on Pearson product-moment correlation coefficient. The affy package in the R statistical software program was used for background correction and normalization of microarray data [18].

### Western blotting

The total protein was extracted using RIPA Lysis Buffer (Beyotime, China) according to the manufacturer’s instructions, supplemented with a protease inhibitor-cocktail (Roche applied science) and PMSF (1 mM). Protein samples (20 μg) were resolved in SDS-PAGE and were transferred to PVDF membranes, and incubated with specific antibodies (anti-Sox4 [Cat. No: LS-C97708, LSBio, 1:1000]; anti-HIF-1α [Cat. No: ab179483, abcam, 1:1000]; anti-β-catenin [Cat. No: ab16051, abcam, 1:4000]. HRP-conjugated goat anti-rabbit IgG antibody [Cat. No: ab205718, abcam, 1:5000] was used as the secondary antibody. Immunoreactive bands were detected using the ECL system (Amersham Pharmacia Biotech). Results were normalized to the expression of GAPDH.

### Quantitative real-time PCR

Total RNA was extracted using TRIzol reagent (Invitrogen). First-strand cDNA was synthesized from 500 ng of total RNA using the PrimeScript RT Reagent Kit (TaKaRa) for detecting mRNA and lncRNA levels. Real-time PCR was performed in triplicate using SYBR Premix Ex Taq (TaKaRa) on CFX96 Real-Time PCR Detection System (Bio-Rad). Expression levels of mRNAs and lncRNAs were normalized to GAPDH and 18S rRNA respectively, and the relative expression of genes was calculated with the 2^-ΔΔCT method. The sequences of primers used here were shown in Table S2.

### In vitro cell viability assay

Cell viability was measured using the Cell Counting Kit-8 assay (CCK-8; 7Sea Biotech, Shanghai, China). Transfected cells (A549 and H1299) were seeded into 96-well plates at a density of 2000 cells/well (*n* = 5 for each time point) in a final volume of 100 μl. CCK-8 solution (10 μl) was added to each well at 24-, 48-, 72-, 96-, and 120-h time points. The absorbance at 450 nm was measured after incubation for 2 h at 37 °C to calculate the number of viable cells.

### Transwell migration assay

Migration assays were performed as described previously [15]. Briefly, 3 × 10^4^ cells suspended in medium without serum or growth factors were plated in the top chamber (24-well insert; pore size, 8 μm; Corning Costar), and medium supplemented with serum was used as a chemoattractant in the lower chamber. The cells were incubated for 24 h and cells that did not migrate through the pores were removed by a cotton swab. Cells on the lower surface of the membrane were stained with crystal violet, air dried and photographed.

### FITC-phalloidin staining

Cells were grown on coverslips, fixed with 4% fresh paraformaldehyde for 15 min at room temperature, permeabilized with 0.25% Triton X-100 in PBS for 10 min, and blocked with 1% bovine serum albumin (BSA) at room temperature for 20 min. These cells were subsequently stained with 5 μg/ml FITC-phalloidin (Sigma-Aldrich, USA) for 1 h at room temperature in the dark, washed, and counterstained with DAPI for 5 min. Confocal microscopy (Nikon A1R) was employed to observe F-actin distribution.

### Luciferase reporter assay

The *CASC15* promoter region (− 988 to + 37 bp surrounding the transcription start site) was cloned by PCR amplification of genomic DNA from 293 T cells and inserted into the pGL3-basic vector to generate the pGL3-CASC15 parental construct. The pGL3-CASC15 mutant construct was made by site-directed mutagenesis of the pGL3-CASC15 vector. The reporter constructs were co-transfected into H1299 cells with different expression vectors and internal control plasmids under normoxic or hypoxic conditions, as described elsewhere [19]. To confirm the effect of endogenous HIF-1α-repression and its influence on CASC15 promoter activity, the same set of plasmids was tested on H1299-siControl and H1299-siHIF-1α cells. Primers used to amplify the *CASC15* promoter region are as follows: pGL3-CASC15-F: 5′-AAGGGTCGGGAGCTGTTCCT-3′, pGL3-CASC15-R: 5′-GGCAGGTTTCTCCCTCTCAC-3′.

For TOP-FLASH reporter assay, TOP-FLASH (TOP) and FOP-FLASH (FOP) plasmids were used. Briefly, A549 cells grown in 24-well plates were transfected in triplicate with a mixture containing 150 ng of firefly luciferase reporter gene construct (either TOP-FLASH or FOP-FLASH reporter plasmid), 150 ng of the SOX4 expression vector (or the corresponding backbone vector), 100 μM si-CASC15 (or si-Control), and 5 ng of the pRL-TK Renilla luciferase construct (for normalization). Cell extracts were prepared 48 h after transfection, and the luciferase activity was measured using the Dual-Luciferase Reporter Assay System (Promega). Data were standardized for each condition by calculating the TOP-FLASH/FOP-FLASH activity ratio.

### Chromatin immunoprecipitation assay

Chromatin immunoprecipitation (ChIP) assays were performed as described previously [20]. Briefly, the cell lysates were incubated with antibody specific for HIF-1α or IgG (as a negative control). The PCR reaction generated a 166-bp product from the CASC15 promoter (− 928 to − 763 bp) containing two adjacent hypoxia response elements (HREs) (− 816 to − 898 bp). A 159-bp product from the VEGF proximal promoter (− 1075 to − 917 bp) was used as a positive control. Primers used in the PCR amplifications of the ChIP assay are as follows: CASC15-ChIP-F: 5′-GAGGGGAAGGAAAGAGGGGA-3′, CASC15-ChIP-R: 5′-AGGCGCCCTCTGACCCTA-3′; VEGF-ChIP-F: 5′-AGTTCCCTGGCAACATCTGG-3′, VEGF-ChIP-R: 5′-GTGTGGTTCCGGGGTTAGT-3′.

### In situ hybridization and immunohistochemistry

In situ hybridization (ISH) and immunohistochemistry (IHC) staining in tissue samples were performed as described previously [20].

### Statistical analysis

Statistical analysis was performed using SPSS 11.0 for Windows. All data were presented as mean ± SEM. The independent Student’s t-test was used to compare the continuous variables between two groups. Overall survival was analyzed with the Kaplan-Meier method and the statistical probability (*p*-value) was generated by log-rank test. Differences were considered significant when *p* < 0.05 (*), *p* < 0.01 (**) or *p* < 0.001 (***).

## Results

### CASC15 is highly expressed in NSCLC and is essential for tumor cell migration and growth

**Fig. 1:**
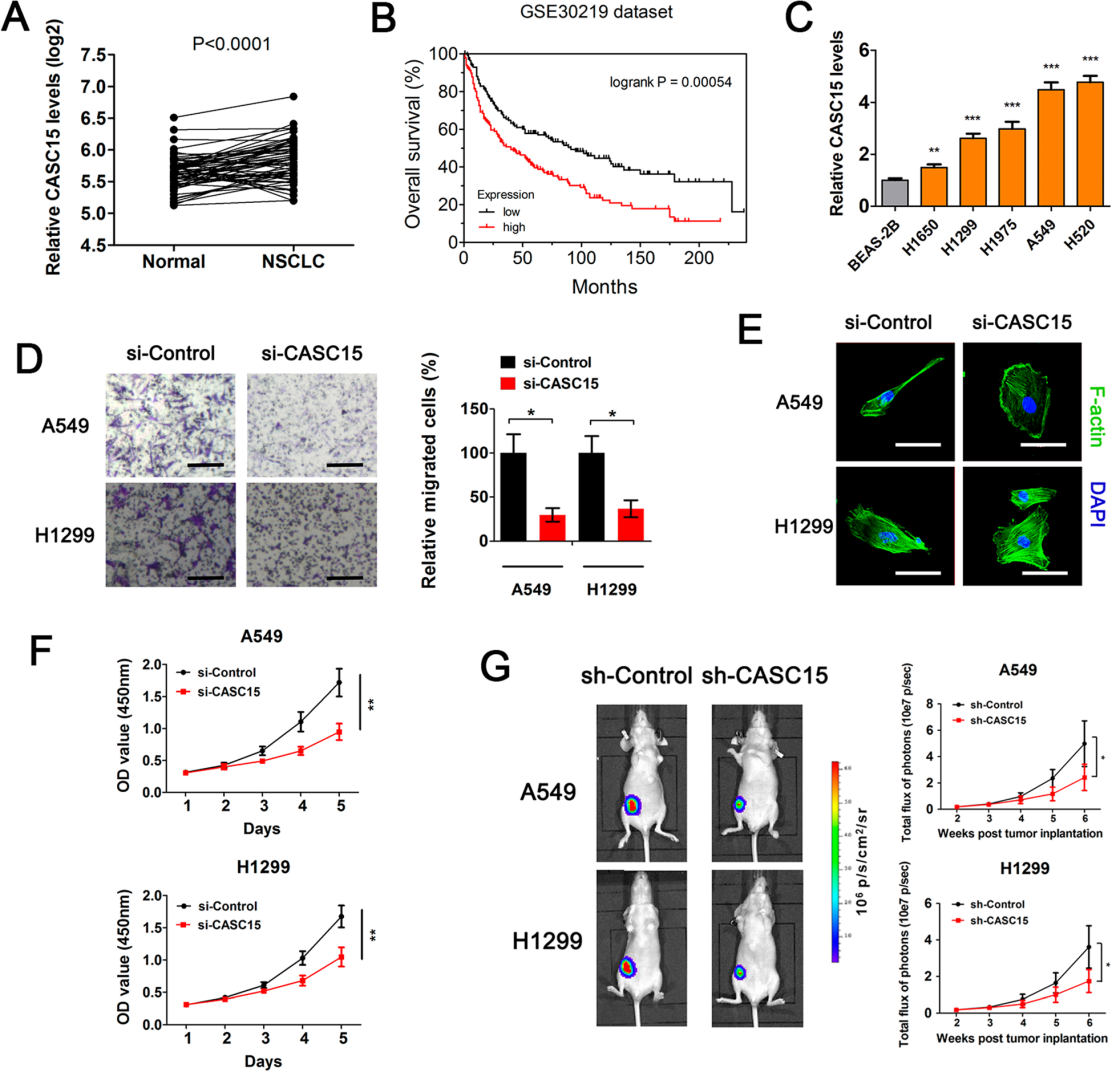
CASC15 is highly expressed in NSCLC and is essential for tumor cell migration and growth. **a** Relative RNA levels of CASC15 in primary human NSCLC tissues and matched adjacent normal tissues. (GEO: GDS3837, *n* = 60 for each group). **b** Kaplan-Meier curves representing the probabilities of overall survival in a cohort of 293 lung cancer patients (GEO: GSE30219) stratified according to the expression levels of CASC15. **c** Relative RNA levels of CASC15 (mean ± SEM, *n* = 3) in five NSCLC cell lines (H1650, H1299, H1975, A549, H520) and one normal human bronchial epithelial cell line BEAS-2B. **d** Transwell migration assay of A549 and H1299 cells treated with si-Control or si-CASC15 for 72 h. **e** FITC-phalloidin staining of A549 and H1299 cells treated with si-Control or si-CASC15 for 72 h, followed by confocal microscopic imaging. Scale bar: 30 μm. **f** Cell proliferation curves of A549 and H1299 cells treated with si-Control or si-CASC15. The experiment was carried out in quintuplicate wells and repeated at least twice. **g** Bioluminescence imaging in human NSCLC xenograft model. A549 or H1299 cells with stable knockdown of CASC15 were injected subcutaneously into nude mice (n = 6 per group). Afterward, primary tumor growth was monitored by regular measurements using a digital caliper

To uncover novel lncRNAs that are involved in the development of lung cancer, we reanalyzed a publicly available dataset involving sixty pairs of human lung carcinomas and adjacent normal lung tissues (GEO dataset GDS3837). Results showed that, CASC15, one lncRNA that was firstly identified as a tumor suppressor in neuroblastoma but was later reported to play an oncogenic role in melanoma [21, 22], was significantly upregulated in NSCLC compared with matched adjacent normal tissues (*p* < 0.001, Fig. 1a). Kaplan-Meier analysis of another dataset involving 293 lung tumor samples (GEO dataset GSE30219) revealed that high expression levels of CASC15 significantly correlated with a reduction in overall survival in NSCLC patients (log-rank test, *p* = 0.00054, Fig. 1b), suggesting that CASC15 upregulation might be crucial in NSCLC tumorigenesis and progression. To investigate whether the expression level of CASC15 is correlated with the malignant phenotype of NSCLC cells in vitro, we cultured five NSCLC cell lines and one normal human bronchial epithelial cell line BEAS-2B. Subsequent quantitative PCR (qPCR) analysis showed that CASC15 were widely overexpressed in NSCLC cell lines (Fig. 1c). Transwell migration assay revealed that siRNA-mediated knockdown of CASC15 resulted in a remarkable suppression of motility of A549 and H1299 cells, reducing the number of migrating cells to about 30% of that in the control group (Fig. 1d and Fig. S1). FITC-phalloidin staining followed by confocal microscopic imaging showed that CASC15 silencing caused an obvious reorganization of F-actin and morphological change from spindle to epithelioid in these two cell lines (Fig. 1e). Moreover, we performed CCK-8 assay and found that CASC15 knockdown led to a dramatic reduction in cell viability and proliferation of A549 and H1299 cells (Fig. 1f). To examine the effect of CASC15 on lung tumor growth in vivo, we established a subcutaneous xenograft model in nude mice using luciferase-expressing A549 and H1299 cells. One week after tumor cell inoculation, tumor growth were monitored by regular measurements using a digital caliper. As expected, stable knockdown of CASC15 significantly inhibited tumor growth in nude mice bearing human lung carcinoma xenografts (Fig. 1g). Taken together, these results supported the notion that CASC15 functions as an oncogenic lncRNA in NSCLC.

### CASC15 exerts tumor-promoting effects in NSCLC cells mainly by upregulating its neighboring oncogene SOX4

**Fig. 2:**
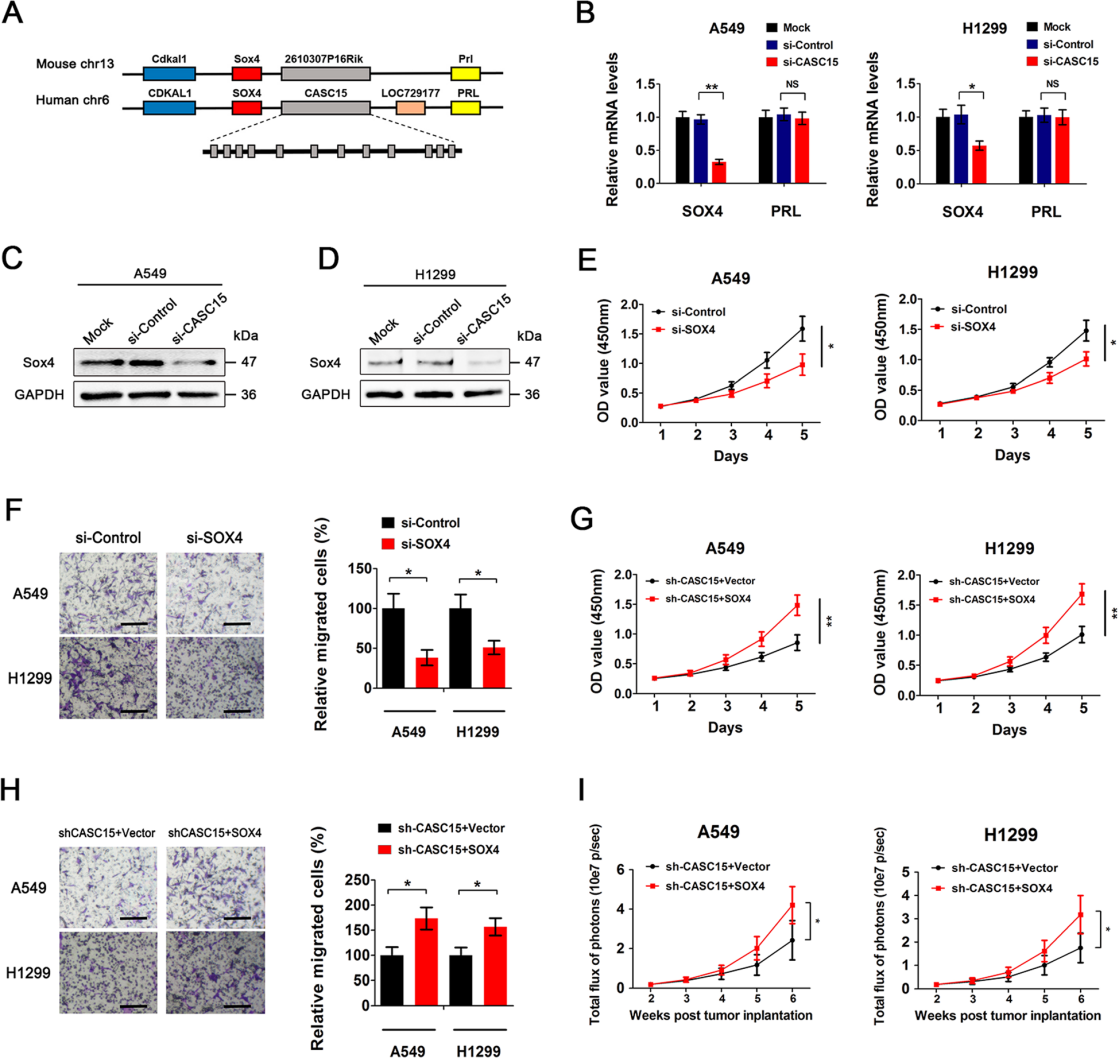
CASC15 exerts tumor-promoting effects in NSCLC cells mainly by upregulating its neighboring oncogene SOX4. **a** Schematic diagram showing the genomic locations of *CASC15* and neighboring genes. The mouse *2610307P16Rik* gene is homologous to the *CASC15* gene in humans. **b** Relative mRNA levels of SOX4 and PRL (mean ± SEM, n = 3) in A549 and H1299 cells treated with si-Control or si-CASC15 for 48 h. (C and D) Western blot analysis of SOX4 protein levels in A549 (**c**) and H1299 (**d**) cells treated with si-Control or si-CASC15 for 48 h. Blots were probed with an antibody against GAPDH to ensure equal loading. **e** Cell proliferation curves of A549 and H1299 cells treated with si-Control or si-SOX4. The experiment was carried out in quintuplicate wells and repeated at least twice. **f** Transwell migration assay of A549 and H1299 cells treated with si-Control or si-SOX4 for 48 h. **g** Cell proliferation curves of A549 and H1299 cells with stable knockdown of CASC15 or with concurrent overexpression of SOX4. The experiment was carried out in quintuplicate wells and repeated at least twice. **h** Transwell migration assay of A549 and H1299 cells with stable knockdown of CASC15 or with concurrent overexpression of SOX4. **i** Bioluminescence imaging in human NSCLC xenograft model. A549 and H1299 cells with stable knockdown of CASC15 or with concurrent overexpression of SOX4 were injected subcutaneously into nude mice (n = 6 per group). Afterward, primary tumor growth was monitored by regular measurements using a digital caliper

The *CASC15* intergenic locus, formerly known as the *FLJ22536* or *LINC00340* locus, spans over 500 kb between the *SOX4* and *PRL* genes on chromosome 6p22.3 (Fig. 2a). To investigate whether CASC15 exerts tumor-promoting effects in NSCLC cells by influencing the expression of neighbouring genes [23], we firstly performed qPCR to analyze the expression levels of SOX4 and PRL upon CASC15 silencing. Results showed that knockdown of CASC15 in NSCLC cells markedly decreased SOX4 mRNA levels but had no effect on PRL expression (Fig. 2b). Further Western blotting analysis confirmed that CASC15 depletion caused significant downregulation of SOX4 at the protein level in both A549 and H1299 cells (Fig. 2c,d). Then, we performed CCK-8 assay and transwell migration assay to test the role of SOX4 in proliferation and migration of NSCLC cells. Knockdown of SOX4 also resulted in a remarkable suppression of proliferation and motility of A549 and H1299 cells (Fig. 2e,f and Fig. S2), to an extent similar to that caused by CASC15 knockdown (Fig. 1d,f). Moreover, ectopic expression of SOX4 largely abrogated the inhibitory effects on cell proliferation and migration mediated by CASC15 knockdown (Fig. 2g,h). In vivo experiments demonstrated that, compared with control groups with stable knockdown of CASC15, concurrent overexpression of SOX4 significantly promoted tumor growth in nude mice bearing human lung carcinoma xenografts (Fig. 2i and Fig. S3). To sum up, these results suggested that CASC15 exerts tumor-promoting effects in NSCLC cells mainly by upregulating its neighboring oncogene SOX4.

### CASC15 activates Wnt signaling in NSCLC via SOX4-mediated stabilization of β-catenin protein

**Fig. 3:**
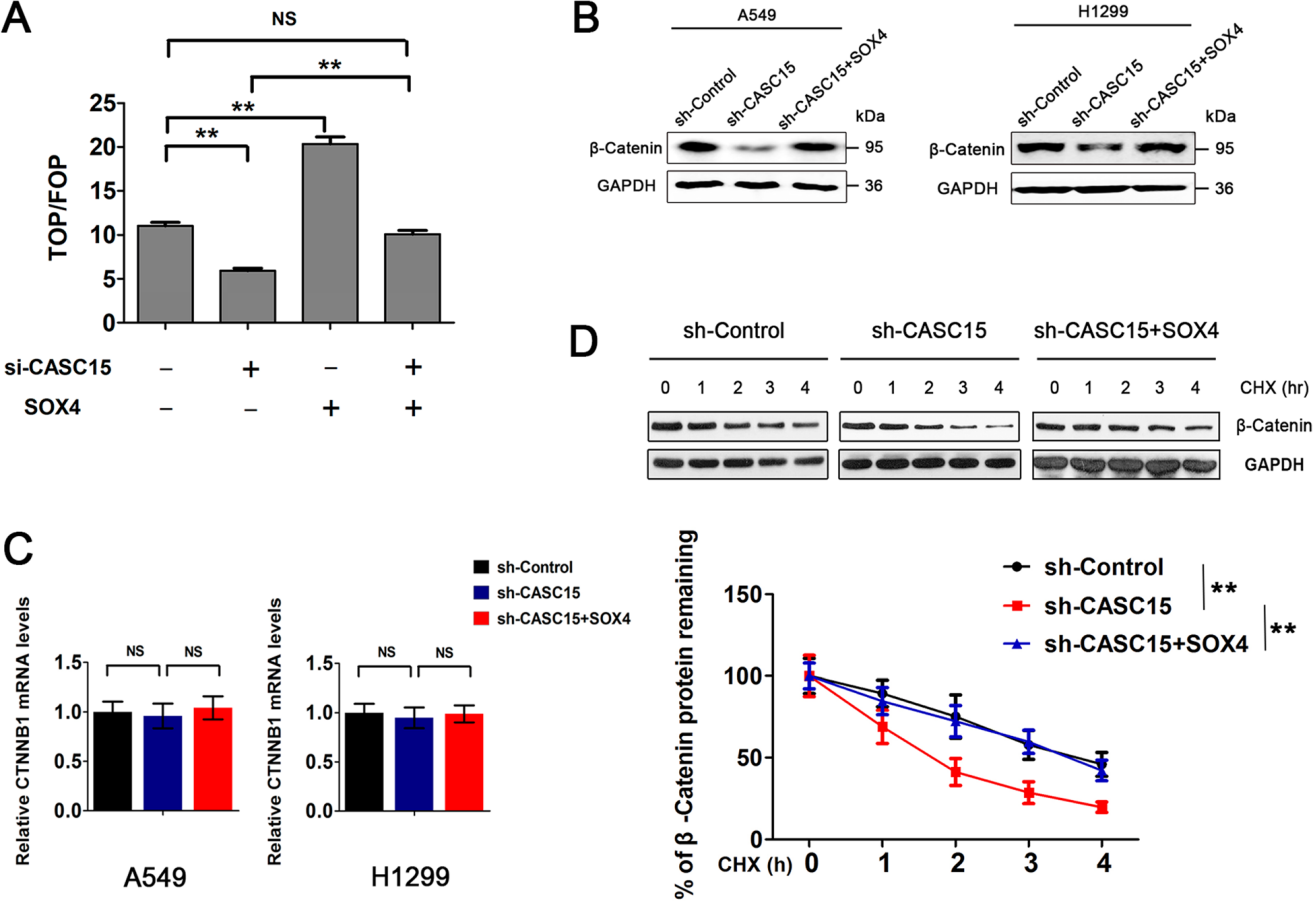
CASC15 activates Wnt signaling in NSCLC via SOX4-mediated stabilization of β-catenin protein. **a** TOP-FLASH assay of A549 cells transfected with si-CASC15 or with concurrent overexpression of SOX4. TOP-FLASH (TOP) or FOP-FLASH (FOP) plasmids were used for the reporter assay. pRL-TK was co-transfected as an internal control. The results are presented as the TOP/FOP ratio. **b** Western blot analysis of β-catenin protein levels in A549 and H1299 cells with stable knockdown of CASC15 or with concurrent overexpression of SOX4. Blots were probed with an antibody against GAPDH to ensure equal loading. **c** Relative mRNA levels of CTNNB1 (mean ± SEM, n = 3) in A549 and H1299 cells with stable knockdown of CASC15 or with concurrent overexpression of SOX4. **d** A549 cells with stable knockdown of CASC15 were transfected with SOX4 expression plasmids or empty vector control. The cell lysates were collected 0, 60, 120, 180, or 240 min after cycloheximide treatment (CHX, 10 μg/ml), and the β-catenin protein levels were detected by Western blotting

Previous studies have identified Wnt-β/catenin pathway as a regulatory target of SOX4 and that SOX4 functions to stabilize β-catenin protein [24, 25]. To determine whether CASC15 could activate Wnt signaling in NSCLC, we measured Wnt signaling activity by using the TOP-FLASH reporter assay in A549 cells, which was reported to express high levels of endogenous β-catenin [26]. Results revealed that β-catenin activity was dramatically reduced by CASC15 knockdown and was rescued by simultaneous overexpression of SOX4 in the same cells (Fig. 3a). Furthermore, Western blotting analysis showed that stable knockdown of CASC15 caused significant downregulation of β-catenin at the protein level in both A549 and H1299 cells, and this effect was largely abrogated by ectopic expression of SOX4 (Fig. 3b). However, CASC15 knockdown did not result in obvious change in β-catenin mRNA levels, regardless of whether or not SOX4 was overexpressed simultaneously (Fig. 3c), suggesting that β-catenin is not regulated by CASC15 or SOX4 at the transcriptional level in NSCLC cells. To investigate the effect of CASC15 on β-catenin protein stability, A549-shCASC15 cells and A549-shControl cells were treated with cycloheximide (CHX) to inhibit protein synthesis and harvested at the indicated time points. Results showed that the β-catenin protein levels decreased overtime in CHX-treated cells and CASC15 silencing significantly accelerated the degradation rate of β-catenin protein (Fig. 3d, upper panel). Meanwhile, ectopic expression of SOX4 in A549-shCASC15 cells extended the half-life of the β-catenin protein to that of A549-shControl cells (Fig. 3d, lower panel). Collectively, these results indicated that CASC15 activates Wnt signaling in NSCLC via SOX4-mediated stabilization of β-catenin protein.

### CASC15 is transcriptionally activated by the hypoxia/HIF-1α signaling in NSCLC

**Fig. 4:**
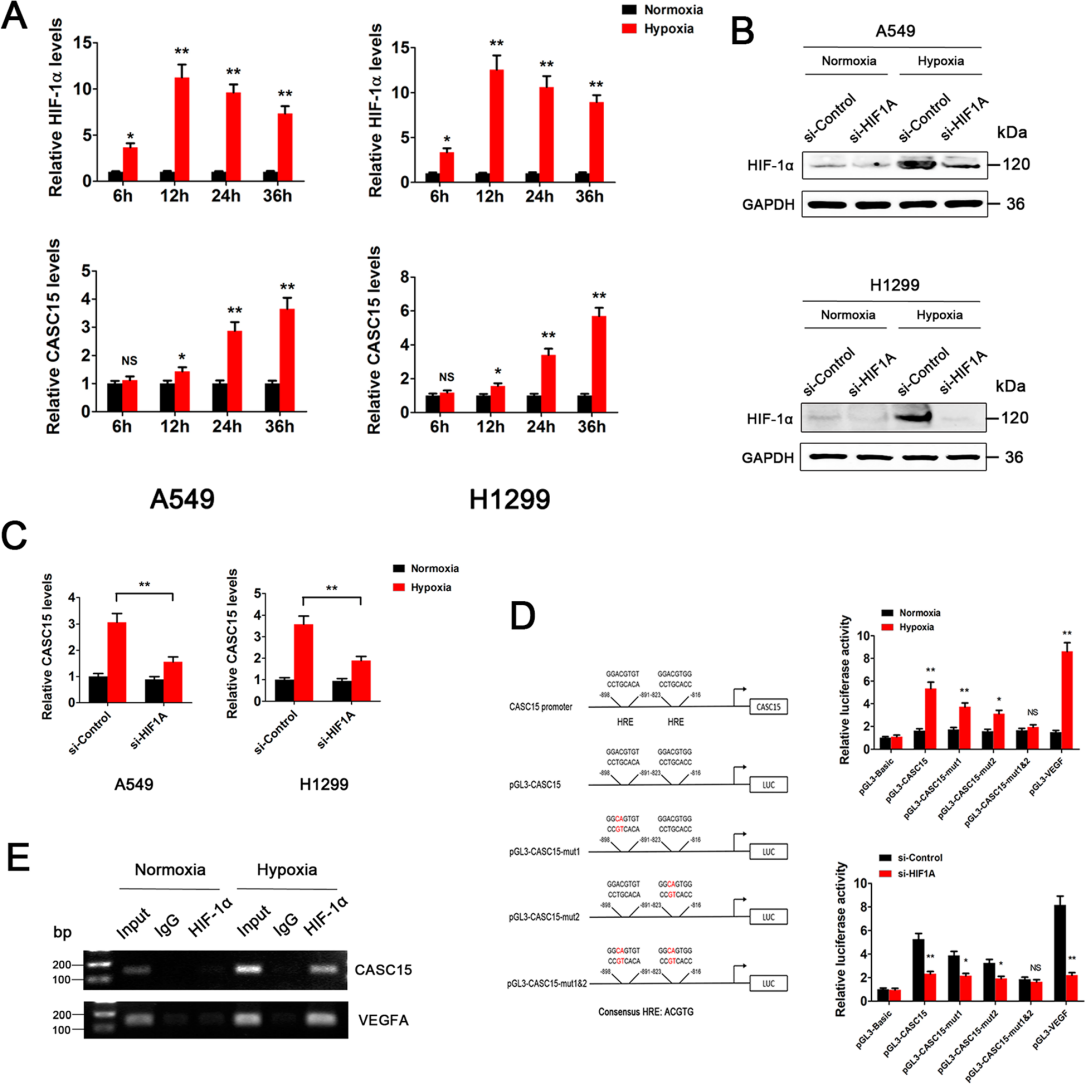
CASC15 is transcriptionally activated by the hypoxia/HIF-1α signaling in NSCLC. **a** Hypoxia induces dynamic changes in the gene expression profiles of HIF-1α and CASC15 in A549 and H1299 cells. Upper panel: the protein levels of HIF-1α were monitored by Western blotting and were normalized to GAPDH protein levels. Data are presented as mean ± SEM from three independent experiments and are expressed as relative fold-change over normoxic controls; Lower panel: the RNA levels of CASC15 were monitored in qRT-PCR experiments and were normalized to 18S expression levels. **b** Western blot analysis of HIF-1α protein levels in A549 and H1299 cells, which were treated with si-Control or si-HIF1A and then subjected to either normoxia or hypoxia for 24 h. **c** qRT-PCR analysis of CASC15 RNA levels in A549 and H1299 cells, which were treated with si-Control or si-HIF1A and then subjected to either normoxia or hypoxia for 24 h. **d** HRE- and HIF-1α-dependent CASC15 transactivation under hypoxia. Left panel: schematic representation of the promoter region of *CASC15* and the reporter constructs used in luciferase reporter assay; Upper right panel: A549 cells were co-transfected with luciferase reporters driven by *CASC15* promoter fragment with wild-type HREs (pGL3-CASC15) or mutated HREs (pGL3-CASC15-mut1, pGL3-CASC15-mut2, and pGL3-CASC15-mut1&2), or VEGF-Luc positive control, or pGL-3 negative control, respectively, along with Renilla-luc reporter. Twenty-four hours post-transfection, cells were subjected to either normoxia or hypoxia for 24 h, followed by luciferase activity assay; Lower right panel: A549 cells were transfected with above constructs respectively, along with Renilla-luc reporter. Twenty-four hours post-transfection, cells were subjected to hypoxia for 24 h, followed by luciferase activity assay. **e** ChIP analysis of A549 cells grown under normoxia or hypoxia for 24 h. Chromatin was incubated with IgG or anti-HIF-1α antibody. The DNA precipitates were subjected to PCR amplification using primer set for *CASC15* promoter (upper panel) or *VEGFA* promoter (lower panel), as positive control

Numerous studies have demonstrated that CASC15 expression is elevated in a variety of human malignancies, such as melanoma, gastric cancer, liver cancer, and acute leukemia [22, 27–29]. However, to date, little is known about the mechanisms governing CASC15 upregulation in cancer. To determine whether the hypoxia/HIF-1α signaling could activate CASC15 expression, NSCLC cells were placed in a 1% oxygen environment for 0 ~ 36 h and the expression levels of HIF-1α and CASC15 were measured. The results showed that HIF-1α expression levels in A549 and H1299 cells were significantly induced upon hypoxia and reached an induction peak at ~ 12 h (Fig. 4a, upper panel). Then, HIF-1α levels gradually decreased but remained elevated throughout the 36-h period. Intriguingly, CASC15 expression started to increase after 12 h of hypoxia and reached a 4 ~ 6-fold induction at 36 h (Fig. 4a, lower panel). These results indicated that CASC15 might be transcriptionally regulated by the hypoxia/HIF-1α signaling in NSCLC cells. To test this hypothesis, we knocked down the expression of HIF-1α in A549 and H1299 cells under either normoxic or hypoxic conditions (Fig. 4b). Subsequent qPCR analysis revealed that hypoxia-induced upregulation of CASC15 was largely eliminated by HIF-1α silencing (Fig. 4c). Moreover, in vivo experiments demonstrated that stable knockdown of HIF-1α significantly decreased the expression level of CASC15 in A549 xenograft tissues (Fig. S4). Thus, we conclude that hypoxia induces CASC15 expression via a HIF-1α-dependent pathway.

To demonstrate whether CASC15 is directly regulated by HIF-1α, two adjacent putative hypoxia-response elements (HREs, also known as HIF-1 binding sites) were identified in the promoter of the *CASC15* gene (Fig. 4d, left panel). Then, luciferase reporters driven by *CASC15* promoter fragment with wild-type HREs (pGL3-CASC15) or mutated HREs (pGL3-CASC15-mut1, pGL3-CASC15-mut2, and pGL3-CASC15-mut1&2) were generated and tested under normoxic or hypoxic conditions (Fig. 4d, left panel). Under normoxic conditions, an approximately two-fold higher luciferase activity than that of the empty vector was detected in both pGL3-CASC15 and pGL3-CASC15-mut, as well as the VEGF-Luc reporter. However, under hypoxic conditions, a nearly six-fold induction of luciferase activity in pGL3-CASC15 construct was observed. Mutation of a single HRE in *CASC15* promoter partially impaired the inductive effect caused by hypoxia, while mutation of both HREs decreased the luciferase activity to nearly basal levels (Fig. 4d, upper right panel). The VEGF-Luc positive control gave rise to a nine-fold induction of luciferase activity. These results strongly suggested that hypoxia-induced CASC15 transactivation is mainly dependent on intact HREs. To further confirm a HIF-1α dependent CASC15 transactivation, we knocked down the expression of HIF-1α and tested luciferase reporter activity in A549 cells under hypoxic conditions. Compared to the empty vector control, luciferase activity of pGL3-CASC15 construct under hypoxic conditions was 5-fold higher in A549-siControl cells, but less than 2.5-fold higher in A549-siHIF1A cells (Fig. 4d, lower right panel). Then, we performed ChIP assays to investigate whether HIF-1α transcription factor binds directly to the *CASC15* promoter. The PCR-amplified fragment, corresponding to a portion of the *CASC15* promoter where HIF-1α bound to, was detected in the input samples (Fig. 4e, upper panel, lanes 2 and 5) or in the sample immunoprecipitated with HIF-1α antibody only under hypoxic conditions (Fig. 4e, upper panel, lane 7), but not under normoxic conditions (Fig. 4e, upper panel, lane 4). The HIF-1α binding to the VEGF-A promoter, serving as positive control, was shown (Fig. 4e, lower panel). Altogether, these results demonstrated that CASC15 is transcriptionally activated by the hypoxia/HIF-1α signaling in NSCLC.

### HIF-1α/CASC15/SOX4/β-catenin pathway is activated in a substantial subset of NSCLC patients

**Fig. 5:**
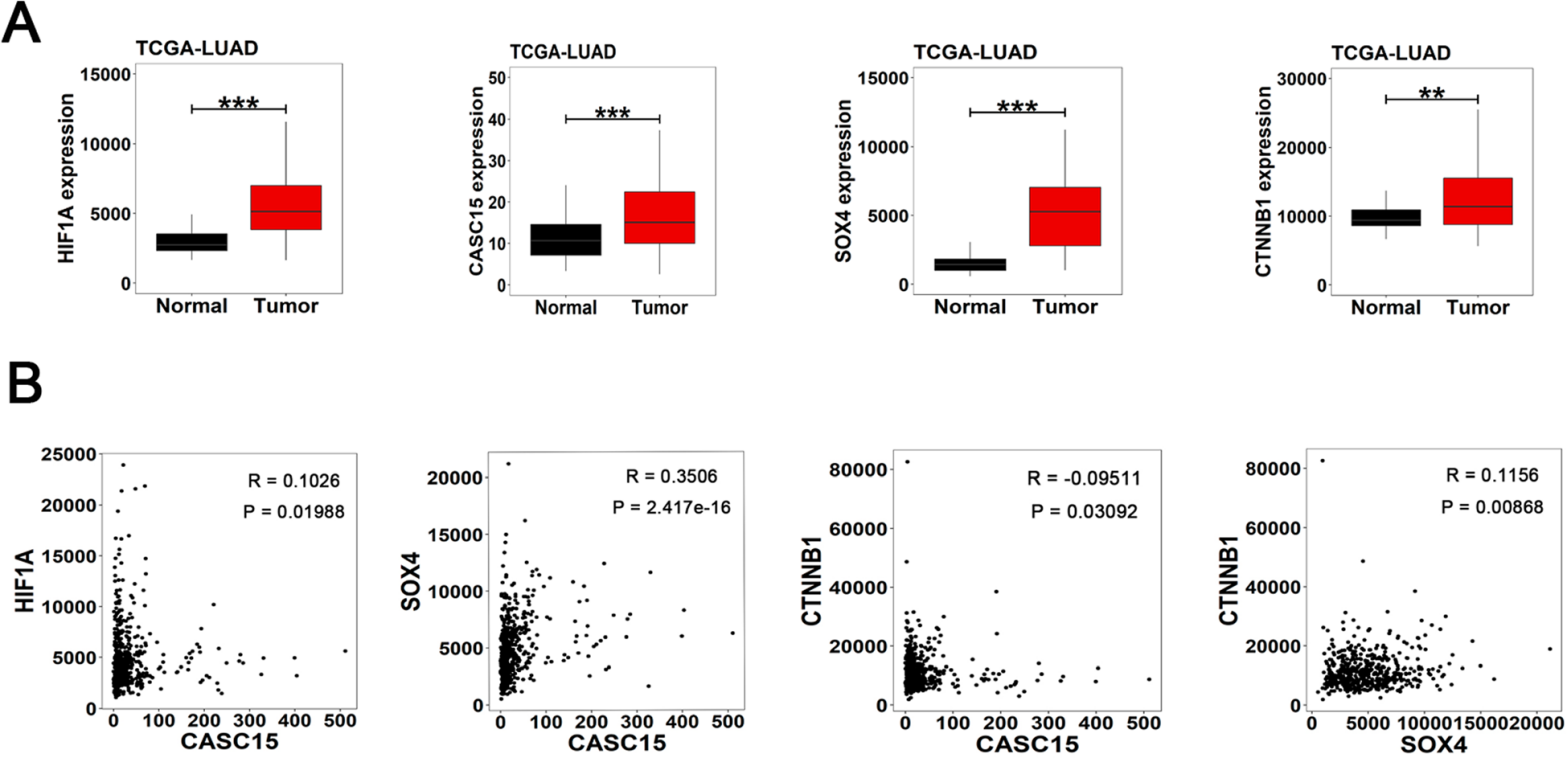
Genes involved in the HIF-1α/CASC15/SOX4/β-catenin axis are highly expressed in NSCLC tissues. **a** Relative RNA levels of HIF1A, CASC15, SOX4, and CTNNB1 in the TCGA-LUAD cohort involving 58 matched pairs of lung adenocarcinoma and adjacent normal lung tissues. **b** Pairwise correlations among CASC15, HIF1A, SOX4 and CTNNB1 expression levels in the TCGA-LUAD cohort containing 515 lung adenocarcinoma tissues

Based on the above evidence, we concluded that lncRNA CASC15 could exert tumor-promoting effects in NSCLC cell lines through a novel HIF-1α/CASC15/SOX4/β-catenin axis. To validate this axis in clinical samples, we downloaded and analyzed the gene expression profiles of HIF1A, CASC15, SOX4, and CTNNB1 (β-catenin) in two lung adenocarcinoma cohorts from TCGA database. The first cohort contains 58 matched pairs of lung adenocarcinoma and adjacent normal lung tissues, and the results showed that all four genes involved in the HIF-1α/CASC15/SOX4/β-catenin axis are highly expressed in cancer tissues compared with normal tissues (Fig. 5a). Then, we performed co-expression analysis in another cohort containing 515 lung adenocarcinoma tissues. We found that positive correlations are particularly strong between HIF1A and CASC15, CASC15 and SOX4, and SOX4 and CTNNB1 (Fig. 5b), indicating potential regulatory relationships between the genes involved in the HIF-1α/CASC15/SOX4/β-catenin pathway.

**Fig. 6:**
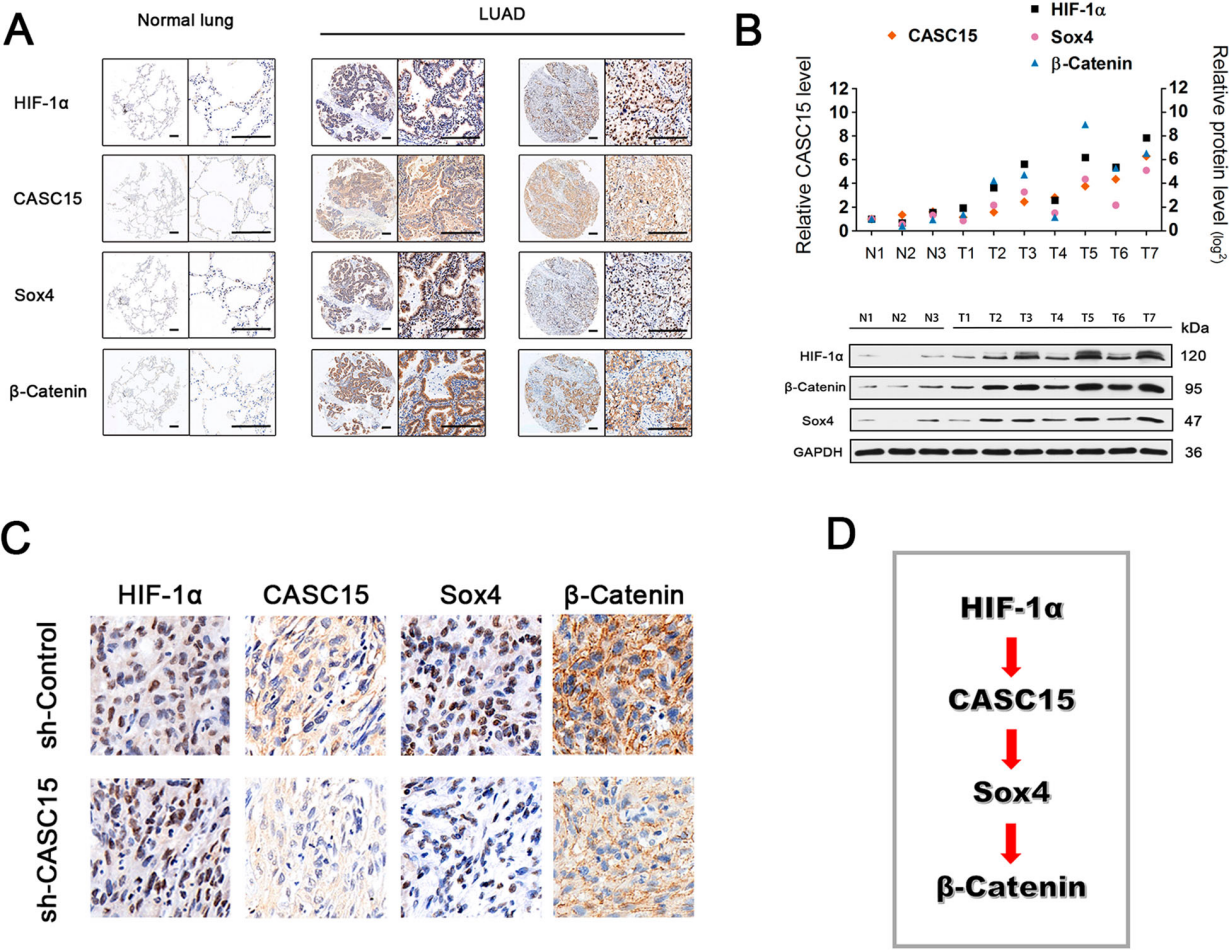
HIF-1α/CASC15/SOX4/β-catenin pathway is activated in a substantial subset of NSCLC patients and A549 xenograft tissues. **a** Representative ISH staining of CASC15 and IHC staining of HIF-1α, SOX4, and β-catenin in a tissue microarray consisting of 35 matched pairs of NSCLC and adjacent normal lung tissues. Scale bar: 200 μm. **b** Positive correlation between CASC15 expression and levels of HIF-1α, SOX4, and β-catenin in NSCLC and adjacent normal lung tissues. LncRNA expression was evaluated by qRT-PCR, and protein abundance was evaluated by Western blotting. **c** Representative ISH staining of CASC15 and IHC staining of HIF-1α, SOX4, and β-catenin in A549-shControl and A549-shCASC15 xenograft tissues. **d** A schematic model of HIF-1α/CASC15/SOX4/β-catenin signaling pathway activated in NSCLC

Next, we performed RNA-ISH and IHC assays to examine the expression levels of lncRNA CASC15 and its related proteins, in A549 xenograft tissues (refer to Fig. 1g) and NSCLC tissue microarrays. As shown in Fig. 6a, HIF-1α, CASC15, SOX4, and β-catenin were aberrantly overexpressed in a substantial proportion of NSCLC patients (45.7%, 16/35), while in normal lung tissues their expressions were nearly absent. Intriguingly, we found that silencing of CASC15 in A549 xenograft tissues remarkably reduced the protein levels of SOX4 and β-catenin, but had no effect on HIF-1α expression (Fig. 6c), supporting our notion that CASC15 functions at an intermediate node in the HIF-1α/CASC15/SOX4/β-catenin signaling axis (Fig. 6d). Furthermore, we examined the expression levels of CASC15 and related proteins in fresh samples from seven lung adenocarcinomas and three normal lung tissues. qPCR and Western blotting analyses showed significant positive correlations between levels of CASC15 and its regulators/targets (Fig. 6b). Taken together, these data demonstrated that the HIF-1α/CASC15/SOX4/β-catenin pathway was activated in a substantial subset of NSCLC patients.

## Discussion

One of the common characteristics of rapidly growing solid tumors is the hypoxia of solid tissues. Therefore, the adaptation of cells to hypoxia and the change of glucose metabolism are the biological basis for the survival of tumor tissues [30]. It has been acknowledged that the early phase of solid tumor growth can be divided into two steps: First, malignant cells form small solid tumors, resulting in tumor hypoxia due to relative lag of vascular growth and rapid proliferation of tumor cells. However, hypoxic tumors are usually small, with a diameter of 2 mm ~ 3 mm. Second, hypoxia triggers fundamental changes in gene expression, leading to neovascularization and tumor growth and metastasis [30].

HIF-1α is the oxygen-regulated subunit of HIF-1, a major transcription factor that mediates adaptive responses to hypoxia. Under normoxic conditions, HIF-1α is rapidly degraded by proteosomes after being targeted for ubiquitination, while reduced degradation results in elevated HIF-1α levels in response to hypoxia [31]. In NSCLC, HIF-1α showed a mixed cytoplasmic/nuclear expression pattern in tumor cells, tumor vessels and tumor-infiltrating macrophages, as well as in areas of metaplasia, while normal lung tissues displayed negative or very weak cytoplasmic staining [32]. Increased HIF-1α expression correlates with metastasis, chemotherapy resistance and poor prognosis in a variety of malignancies including NSCLC [31, 33, 34]. Previous studies have confirmed that there are more than 100 target genes transcriptionally regulated by HIF-1α, mainly including genes encoding angiogenesis-related factors and proliferation-related proteins [31]. In recent years, lncRNAs have also been reported to be involved in the hypoxia-related cancer process, implying their potential roles in enabling malignant cells to survive and in promoting tumor development under hypoxic stress. For example, hypoxia-induced histone deacetylase 3 represses lncRNA-LET by reducing the histone acetylation-mediated modulation of the lncRNA-LET promoter region, which leads to the stabilization of nuclear factor 90 protein and hypoxia-induced cancer cell invasion [35]. Another study demonstrated that linc-RoR is a hypoxia-responsive lncRNA that is functionally linked to hypoxia signaling in hepatocellular carcinoma through a miR-145/HIF-1α signaling module [36]. In pancreatic cancer, lncRNA-BX111887 was reported to be induced by HIF-1α in response to hypoxia. lncRNA-BX111887 upregulation contributes to the hypoxia-induced EMT of cancer cells by regulating expression of ZEB1 and its downstream genes E-cadherin and MMP2 [37]. However, while it has been widely accepted that complex coding/noncoding gene regulatory networks take part in the hypoxia-associated tumor progression, it remains largely elusive how HIF-1α/lncRNA axes participate in the development of lung cancer [38].

Here we report that a lncRNA, named CASC15, is transcriptionally activated by the hypoxia/HIF-1α signaling and promotes SOX4 expression in a *cis*-acting manner in NSCLC. CASC15 was firstly identified as a pivotal tumor suppressor in neuroblastoma but was later reported to play an oncogenic role in many other cancer types, such as melanoma, gastric cancer, liver cancer, lung cancer, and acute leukemia [21, 22, 27–29, 39, 40]. Nevertheless, these studies did not uncover the signaling pathways that might activate the expression of CASC15. In this work, we demonstrated that HIF-1α expression levels were significantly induced upon hypoxia and reached an induction peak at about 12 h in NSCLC cells (Fig. 4a, upper panel), which is in accordance with previous reports [41]. Intriguingly, CASC15 started to increase after 12 h of hypoxia, the time point when HIF-1α was highly activated (Fig. 4a, lower panel). This phenomenon nicely supported our notion that CASC15 is transcriptionally activated by HIF-1α. Mechanistic study revealed that two adjacent HREs located in the *CASC15* promoter are indispensable for hypoxia-induced CASC15 upregulation (Fig. 4).Moreover, we also found that the expression levels of CASC15 in NSCLC cells were significantly higher than those in normal human bronchial epithelial cell line BEAS-2B under normoxic conditions (Fig. 1c). This result indicates that CASC15 could be upregulated by other factors besides hypoxia, such as copy number alterations (CNAs) and epigenetic regulators [22, 42]. However, the expression levels of CASC15 could be further upregulated by hypoxia signaling, although the basal expression levels of CASC15 in NSCLC cells were already high (Fig. 4a). To our knowledge, this is the first study to evaluate the molecular crosstalk between CASC15 and hypoxia signaling, which adds a new layer of complexity and possibilities to the regulation of CASC15 expression.

In the present work, we used RNAi to interrogate loss-of-function phenotypes for genes of interest and found that CASC15 knockdown caused significant downregulation of SOX4 in NSCLC cells (Fig. 2). Meanwhile, we performed gain-of-function experiments using lentivirus-mediated overexpression of CASC15 in NSCLC cells. However, results showed that overexpression of CASC15 had no effect on SOX4 expression levels (Fig. S5). In addition, genome analysis revealed that the *CASC15* and *SOX4* loci were adjacent to each other on chromosome 6p22.3 (Fig. 2a). Based on these findings, we speculated that CASC15 might function as an enhancer RNA to activate neighboring *SOX4* genes, and that exogenous CASC15 could not accurately target its genomic locus to regulate transcription of *SOX4* gene. Further studies demonstrated that immunoprecipitation of endogenous WDR5 from NSCLC cell lines specifically retrieved endogenous CASC15 RNA (Fig. S6), supporting our hypothesis that CASC15 RNA might contribute to transcriptional activation of neighboring gene by recruiting WDR5 protein [43]. Moreover, we found that CASC15 functions to stabilize β-catenin protein by upregulating SOX4 (Fig. 3), thereby enhancing the proliferation and migration of NSCLC cells. In line with this, other groups have recently reported that CASC15 could promote tumorigenesis by activating Wnt//β-catenin signaling pathway in colon cancer and melanoma, suggesting that CASC15/β-catenin axis has an important and universal function in cancer development and progression [44, 45].

## Conclusions

HIF-1α/CASC15/SOX4/β-catenin axis plays an essential role in the development and progression of NSCLC. CASC15 was significantly upregulated in NSCLC tissues compared with matched adjacent normal tissues. Silencing of CASC15 in NSCLC cells could lead to a remarkable suppression of tumor cell migration and growth. The present work provides new evidence that lncRNA CASC15 holds great promise to be used as novel biomarkers for NSCLC. Blocking the HIF-1α/CASC15/SOX4/β-catenin axis can serve as a potential therapeutic strategy for treating NSCLC.

## Supplementary Information

**Additional file 1: Figure S1.** qRT-PCR analysis of CASC15 RNA levels in A549 and H1299 cells, which were treated with si-Control or si-CASC15 for 48 hours. **Figure S2.** Western blot analysis of SOX4 protein levels in A549 and H1299 cells, which were treated with si-Control or si-SOX4 for 48 hours. **Figure S3**. Tumor volume in nude mice injected with A549 and H129 cells with stable knockdown of CASC15, or concurrent overexpression of SOX4. **Figure S4.** Representative IHC staining of HIF-1α and ISH staining of CASC15 in A549-shControl and A549-shHIF1A xenograft tissues. **Figure S5.** Western blot analysis of SOX4 protein levels in CASC15-overexpressing H1299 cells and control cells. **Figure S6.** RNA-IP assay detecting potential interactions between CASC15 RNA and WDR5 protein in A549 and H1299 cells. U1 snRNA, which was reported not binding to WDR5, was used as a negative control. **Table S1.** The characteristics of 35 NSCLC patients included in the tissue microarray in our study. **Table S2.** Primer sequences for qRT-PCR.

## Data Availability

All data are fully available without restrictions.

## Abbreviations

CASC15: Cancer susceptibility candidate 15
ChIP: Chromatin immunoprecipitation
CHX: Cycloheximide
GEO: Gene Expression Omnibus
HIF-1α: Hypoxia-inducible factor 1α
HREs: Hypoxia response elements
IHC: Immunohistochemistry
ISH: In situ hybridization
lncRNA: Long non-coding RNA
NSCLC: Non-small cell lung cancer;
qRT-PCR: Quantitative real-time PCR
SOX4: SRY-box transcription factor 4
TCGA: The Cancer Genome Atlas
