# Supplementary material for: Hypoxia-sensitive long noncoding RNA CASC15 promotes lung tumorigenesis by regulating the SOX4/β-catenin axis
Source: J Exp Clin Cancer Res. 2021 Jan 6;40:12. doi: 10.1186/s13046-020-01806-5 (PMC7789733; doi:10.1186/s13046-020-01806-5)
Supplement: Supplementary file 1 — Additional file 1: Figure S1. qRT-PCR analysis of CASC15 RNA levels in A549 and H1299 cells, which were treated with si-Control or si-CASC15 for 48 hours. Figure S2. Western blot analysis of SOX4 protein levels in A549 and H1299 cells, which were treated with si-Control or si-SOX4 for 48 hours. Figure S3. Tumor volume in nude mice injected with A549 and H129 cells with stable knockdown of CASC15, or concurrent overexpression of SOX4. Figure S4. Representative IHC staining of HIF-1α and ISH staining of CASC15 in A549-shControl and A549-shHIF1A xenograft tissues. Figure S5. Western blot analysis of SOX4 protein levels in CASC15-overexpressing H1299 cells and control cells. Figure S6. RNA-IP assay detecting potential interactions between CASC15 RNA and WDR5 protein in A549 and H1299 cells. U1 snRNA, which was reported not binding to WDR5, was used as a negative control. Table S1. The characteristics of 35 NSCLC patients included in the tissue microarray in our study. Table S2. Primer sequences for qRT-PCR. [file 13046_2020_1806_MOESM1_ESM.docx]

**Supplementary Information**

**Supplemental Figure legends**

**Figure S1.** qRT-PCR analysis of CASC15 RNA levels in A549 and H1299 cells, which were treated with si-Control or si-CASC15 for 48 hours.

**Figure S2.** Western blot analysis of SOX4 protein levels in A549 and H1299 cells, which were treated with si-Control or si-SOX4 for 48 hours.

Figure S3. Tumor volume in nude mice injected with A549 and H129 cells with stable knockdown of CASC15, or concurrent overexpression of SOX4.

**Figure S4.** Representative IHC staining of HIF-1α and ISH staining of CASC15 in A549-shControl and A549-shHIF1A xenograft tissues.

**Figure S5.** Western blot analysis of SOX4 protein levels in CASC15-overexpressing H1299 cells and control cells.

**Figure S6.** RNA-IP assay detecting potential interactions between CASC15 RNA and WDR5 protein in A549 and H1299 cells. U1 snRNA, which was reported not binding to WDR5, was used as a negative control.

**Table S1.** The characteristics of 35 NSCLC patients included in the tissue microarray in our study.

**Table S2.** Primer sequences for qRT-PCR.

**Figure S1**

**

**

**Figure S2**


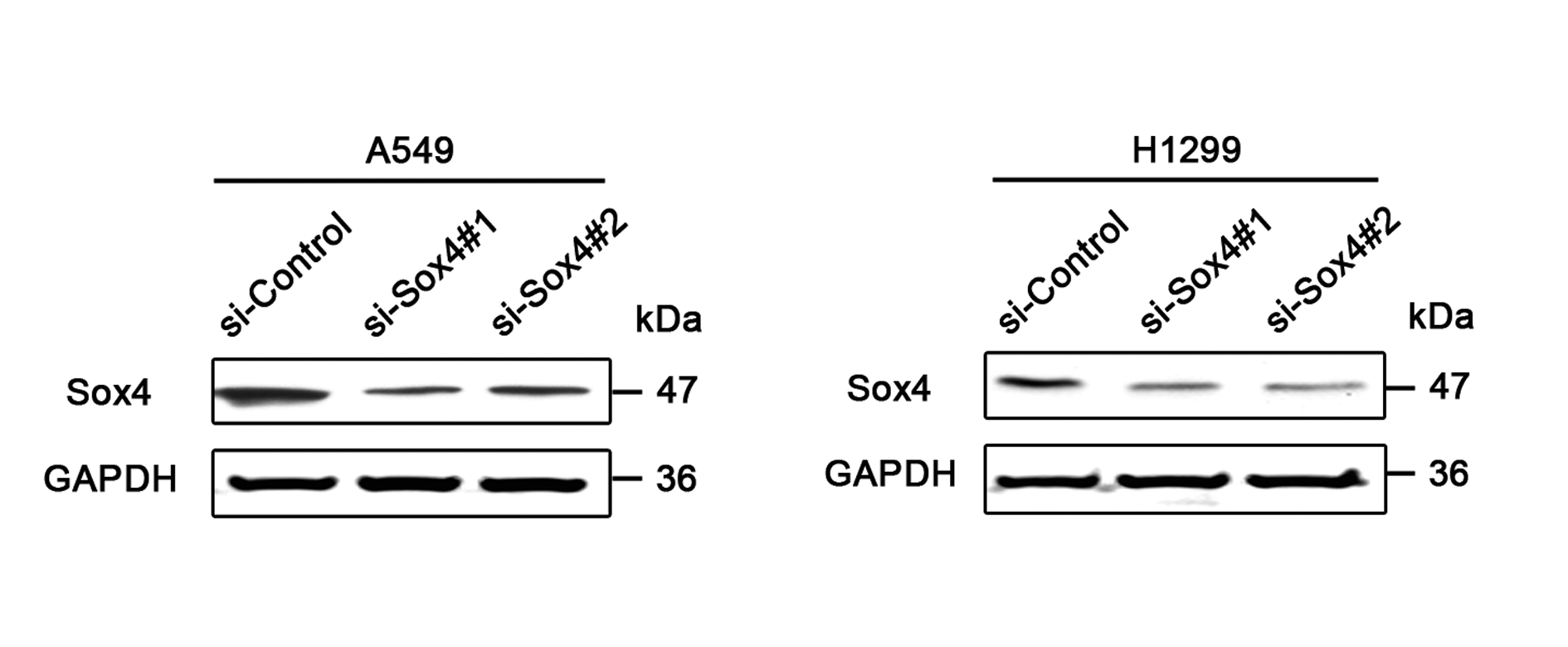


**Figure S3**





**Figure S4**


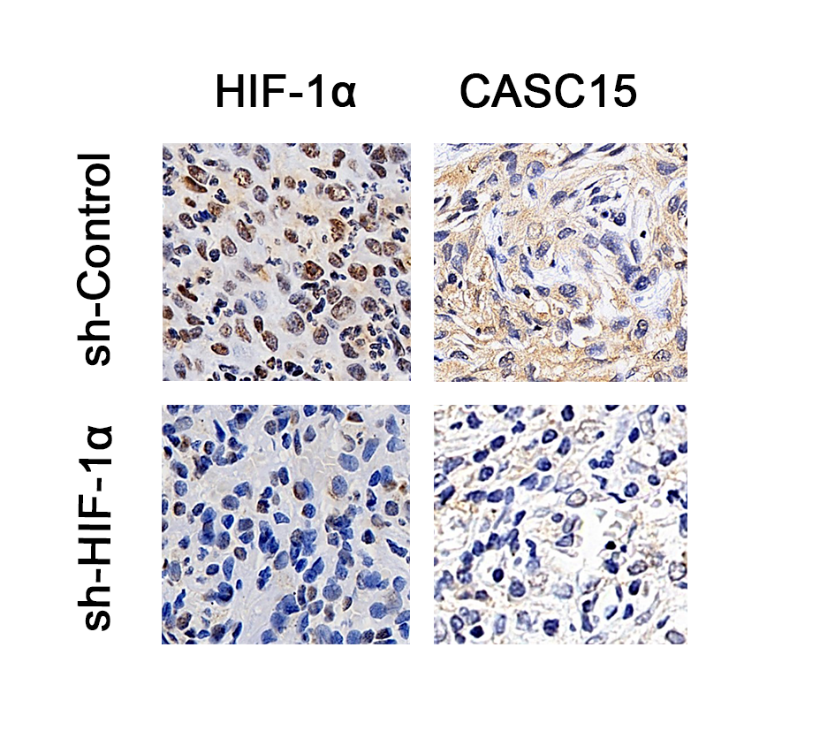


**Figure S5**


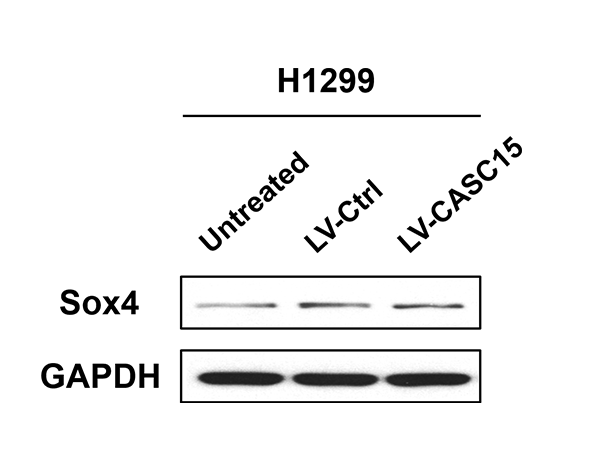


**Figure S6**

**

**

**Table S1**

| Characteristics | Case (n, %) |
| --- | --- |
| **Age** (years) | Media: 60; Range: 47-74 |
| < 60 | 17 (48.6) |
| ≥ 60 | 18 (51.4) |
| **Gender** |  |
| Male | 20 (57.1) |
| Female | 15 (42.9) |
| **Pathologic type** |  |
| Adenocarcinoma | 19 (54.3) |
| Squamous cell carcinoma | 11 (31.4) |
| Others | 5 (14.3) |
| **Differentiated degree** |  |
| Well | 8 (22.9) |
| Media | 15 (42.9) |
| Poor | 12 (34.3) |
| **T stage** |  |
| I-II | 25 (71.4) |
| III-IV | 10 (28.6) |
| **Tumor size** |  |
| < 3 cm | 8 (22.9) |
| ≥ 3 cm | 27 (77.1) |
| **Lymph nodes metastasis** |  |
| No | 24 (68.6) |
| Yes | 11 (31.4) |

**Table S2**

| Primer Sequences for qRT-PCR | | |
| --- | --- | --- |
| Gene | Strand | Sequences |
| CASC15 | forward | 5’-TTAGGGAAAGCCTTCTTTAGGGAT-3’ |
|  | reverse | 5’-CTCCCAGCCCCTATTCCTTT-3’ |
| HIF1A | forward | 5’-TTTTTCAAGCAGTAGGAATTGGA-3’ |
|  | reverse | 5’-GTGATGTAGTAGCTGCATGATCG-3’ |
| SOX4 | forward | 5’-CCAAATCTTTTGGGGACTTTT-3’ |
|  | reverse | 5’-CTGGCCCCTCAACTCCTC-3’ |
| CTNNB1 | forward | 5’-ACAGGGAAGACATCACTGAGCC-3’ |
|  | reverse | 5’-CAGTGGGATGGTGGGTGTAAGA-3’ |
| 18S rRNA | forward | 5’-GTAACCCGTTGAACCCCATT-3’ |
|  | reverse | 5’-CCATCCAATCGGTAGTAGCG-3’ |
| GAPDH | forward | 5’-TCGGAGTCAACGGATTTGGT-3’ |
|  | reverse | 5’-TCGCCCCACTTGATTTTGGA-3’ |
